# Natural Polymorphic Variants in the CYP450 Superfamily: A Review of Potential Structural Mechanisms and Functional Consequences

**DOI:** 10.3390/ijms26167797

**Published:** 2025-08-12

**Authors:** Rafał Prost, Wojciech Płaziński

**Affiliations:** 1 Independent Researcher, 20-075 Lublin, Poland; rafalprost@gmail.com; 2 Jerzy Haber Institute of Catalysis and Surface Chemistry, Polish Academy of Sciences, 30-239 Krakow, Poland; 3 Department of Biopharmacy, Medical University of Lublin, 20-093 Lublin, Poland

**Keywords:** polymorphism, CYP450, cytochrome enzymes, mutations, structural mechanisms, pharmacogenetics

## Abstract

Cytochrome P450 (CYP450) enzymes play an essential role in the metabolism of drugs, particularly in phase I metabolic reactions. In this article, we present a comprehensive review of fifteen selected enzymes belonging to the CYP450 family. The enzymes included in this analysis are CYP7A1, CYP3A4, CYP3A5, CYP2D6, CYP2E1, CYP2C8, CYP2C18, CYP2C9, CYP2C19, CYP2B6, CYP2A6, CYP2A13, CYP1B1, CYP1A1, and CYP1A2. We examined the influence of natural, polymorphic variations within their primary amino acid sequences on their enzymatic function and mechanisms of action. To begin, we compiled a dataset of naturally occurring polymorphic variants for these enzymes. This was achieved through a detailed analysis of entries in the UniProt database, as well as an extensive review of the current scientific literature. For each variant, we included commentary regarding its potential impact on enzyme activity or drug response, based on evidence observed in in vitro experiments, in vivo studies, or clinical trials. Particular emphasis was placed on how such polymorphisms might alter the metabolism of xenobiotics, thereby potentially affecting pharmacological outcomes. In this respect, the work represents the first comprehensive source in the scientific literature that systematically gathers and organizes data on CYP450 polymorphisms, including an assessment of their potential significance in processes mediated by these enzymes. A more detailed comparison of the polymorphism-related in vitro studies is devoted to CYP3A4, an enzyme that displays the largest fraction of clinically significant polymorphs. Secondly, we aimed to establish possible molecular explanations for why specific polymorphisms exhibit clinically or experimentally observable effects. To explore this, we performed a qualitative structural analysis of the enzymes, focusing on shared structural characteristics among the examined members of the CYP450 family. The results of this analysis demonstrate that there is no single universal mechanism by which polymorphisms influence the function of CYP450 enzymes. Instead, the mechanisms vary and may include alterations in the orientation of the enzyme within the lipid membrane, changes affecting the association or dissociation of substrates and products at the active site, structural stabilization or destabilization of the enzyme’s reactive centers, modifications in the way the enzyme interacts with its ligand, or alterations in the character of the interface involved in contact with its redox partner (electron transfer protein). Furthermore, among the polymorphisms that significantly impact enzyme function, mutations involving the substitution of arginine residues for other amino acids appear to be overrepresented.

## 1. Introduction

Cytochrome P450 (CYP450) enzymes play a crucial role in drug metabolism, particularly in phase I metabolic reactions [1,2,3]. All members of this enzyme superfamily are designated with the CYP prefix, followed by a unique identifier, such as 1A1, 2D6, and so on. Specific mutations in the primary amino acid sequence of these enzymes can alter their metabolic activity toward xenobiotics, particularly in terms of the rate of phase I reactions, which is typically expressed through parameters such as the maximum reaction velocity or the Michaelis–Menten constant [4,5,6]. A wide array of pharmacogenetic research is being carried out globally. This includes both in vitro studies [7,8], where various naturally occurring polymorphisms are examined in controlled experimental settings, and clinical trials [9,10,11,12] that explore the pharmacokinetics of xenobiotics in patients with different genetic variants. These studies have contributed to the development of pharmacogenetic guidelines by several independent initiatives, such as the Ubiquitous Pharmacogenetics Consortium and the Clinical Pharmacogenetics Implementation Consortium [13,14].

Despite a general scientific consensus on the relevance of polymorphic variations within the CYP450 family, as well as the dynamically developing field of science relying on studying various polymorphic changes in the context of enzymes’ action, the existing literature lacks a systematic compilation of reported natural polymorphism of CYP450, along with information on their potential biological significance. Furthermore, there is an absence of cross-sectional analyses linking these documented polymorphic changes with structural features of CYP450 enzymes, which are highly conserved within the family. As a result, it remains unclear whether the observed functional impacts of these polymorphisms described in the literature stem from similar structural mechanisms or entirely different ones. This study aimed to fill these gaps and organize the current knowledge regarding polymorphisms within the CYP450 enzyme family.

In this paper, we present a literature review focused on the functional effects of polymorphisms found in selected CYP450 enzymes. Particular emphasis is placed on polymorphisms with documented relevance to the mechanisms of action of these enzymes, based on evidence from experimental (in vitro and in vivo) studies and clinical observations. The enzymes selected for analysis include CYP7A1, CYP3A4, CYP3A5, CYP2D6, CYP2E1, CYP2C8, CYP2C18, CYP2C9, CYP2C19, CYP2B6, CYP2A6, CYP2A13, CYP1B1, CYP1A1, and CYP1A2. This selection was based both on the functional importance and prevalence of these enzymes in scientific research and on the available reports concerning polymorphisms identified within them. This includes both the discovery of novel natural polymorphisms and the presence of direct or indirect evidence suggesting the relevance of these variants for enzyme function [15,16,17].

The paper is divided into two parts. In the first part, we present a compilation of natural polymorphisms observed in selected CYP450 enzymes based on the UniProt database and the associated literature. Additionally, a more detailed comparison of the in vitro studies focuses on the case of the CYP3A4 enzyme, which exhibits the largest fraction of clinically significant variations. In the second part, we attempt to link the information summarized in the first section with available structural data, with the aim of identifying molecular explanations for the physiological consequences of the observed polymorphisms.

## 2. Polymorphisms of CYP450

### 2.1. Natural Polymorphisms of the CYP450 Family and Their Consequences

In this section, we analyze 14 out of the 15 CYP450 enzymes mentioned in the introduction, i.e., CYP7A1, CYP3A4, CYP3A5, CYP2D6, CYP2E1, CYP2C8, CYP2C18, CYP2C9, CYP2C19, CYP2B6, CYP2A6, CYP2A13, CYP1A1, and CYP1A2. The enzyme CYP1B1, although present in the context of the list of existing polymorphisms, was excluded from the phase of the comparison focused on physiological effects. This decision was made due to the distinct role of this enzyme in the human body, including its unique pattern of tissue expression compared with the other enzymes discussed here, and due to the known pathological impact of its mutations. Specifically, mutations in CYP1B1 are linked to primary congenital glaucoma, a serious developmental disorder affecting the eyes. A more detailed examination of the polymorphic variants of this enzyme, and their implications for its mechanism of action and physiological outcomes, is planned for inclusion in a forthcoming publication.

Table 1 contains the core compilation of natural polymorphism enzymes considered in this section. For each variant (except for the case CYP450 1B1), the table includes indications of confirmed functional significance, where available, along with the corresponding references to the relevant scientific literature. When no literature reference is given, the variant presence relied solely on the UniProt record. The data for CYP450 1B1 provide general information on the existence of particular polymorphs, while an in-depth description of their potential significance and mechanism of action is deferred to future work.

**Table 1:** Naturally occurring variants of the enzymes belonging to the CYP450 family and their influences on enzyme function. **Bolded font** in the second column marks the polymorphs of confirmed relevance in either in vitro, in vivo, or clinical studies. * denotes a stop codon.

Enzyme	Mutation	Literature Information	References
CYP1A1	G45D	Novel	[2,18]
	I78T	Novel	[2,18]
	R93W	Novel	[2]
	T173R	Novel	[2]
	R279W	Novel	[19]
	M331I	0.8% frequency	[20]
	I448V	Mutation causing premature stop codone	[21]
	**T461N**	May be partially associated with a higher risk of estrogen-induced cancer	[2,18,22,23]
	**I462V**	May be partially associated with higher risk of estrogen-induced cancer. Closely linked with lung-cancer-susceptibility genotype in CYP1A1.	[18,22,24]
	R464C	Novel	[21]
	R464S	1.7% frequency	[20]
	R477W	Novel	[21]
	V482M	Novel	[2]
	P492R	Novel	[2,21]
CYP1A2	S18C	Novel	[2,18]
	**F21L**	The efficiency of liver activity measured in the caffeine test was not significantly higher than mean (3.5% to 3.12%)	[25]
	P42R	Novel	[26]
	G73R	-	-
	T83M	Novel	[27]
	D104N	-	-
	L111F	-	-
	E168Q	Novel	[27]
	F186L	Novel, suggested to be critical for catalytic activity	[27]
	F205V	-	-
	S212C	Novel	[27]
	R281W	-	-
	S298R		[2]
	G299S	Novel	[27]
	I314V		[2]
	D348N	Novel	[28,29]
	R377Q	Novel	[26]
	I386F	Novel	[28,29]
	C406Y	Novel	[28,29]
	**R431W**	Critical for the tertiary structure of the protein (no holoenzyme was detected for this substitution)	[2,28,29]
	T438I	Novel	[27]
	R456H	Novel	[26]
CYP1B1	S28W	-	-
	R48G	-	-
	P52L	-	-
	W57 *	-	-
	W57C	-	-
	P58*(Q)	-	-
	L59*(P)	-	-
	G61E	-	-
	L77P	-	-
	Y81N	-	-
	A115P	-	-
	A119S	-	-
	M132R	-	-
	Q144P	-	-
	Q144R	-	-
	145	-	-
	A179*del*	-	-
	Q184S	-	-
	A189P	-	-
	D192V	-	-
	P193L	-	-
	V198I	-	-
	N203S	-	-
	S206N	-	-
	S215I	-	-
	E229K	-	-
	G232R	-	-
	F261L	-	-
	R266L	-	-
	SNF269-271*del*	-	-
	FL276-277 *	-	-
	R290*del*	-	-
	V320L	-	-
	A330S	-	-
	L343*del*	-	-
	L345F	-	-
	R355*del*	-	-
	RV355*del*	-	-
	V365M	-	-
	G365W	-	-
	R368H	-	-
	D374N	-	-
	P379L	-	-
	E387K	-	-
	A388T	-	-
	R390C	-	-
	R390H	-	-
	R390S	-	-
	I399S	-	-
	T404*del*	-	-
	V409F	-	-
	V422G	-	-
	N423Y	-	-
	L432V	-	-
	P437L	-	-
	A443G	-	-
	R444Q	-	-
	F445C	-	-
	N453S	-	-
	G466D	-	-
	R469W	-	-
	E499G	-	-
	S515L	-	-
	V518A	-	-
	R523T	-	-
	D530G	-	-
CYP2A6	G5R	Novel	[2,30]
	M6I	-	-
	L20F	-	-
	S29N	Novel	[2,30,31]
	Q53H	-	-
	R64H	-	-
	V68M	-	-
	V79M	-	-
	R101Q	-	-
	E103K	-	-
	V116M	-	-
	F118L	Novel	[2,32]
	R128L	Novel	[32]
	R128Q	Novel	[2,33]
	S131A	Novel	[32]
	R148C	-	-
	D158E	-	-
	K194E	Novel	[30]
	**R203C**	Reduced activity towards C-oxidation of nicotine, more frequent in non-smokers	[34]
CYP2A13	R25Q	Novel	[35,36]
	R101Q	Novel	[36]
	T134TT (duplication)	Novel	[36]
	D158E	Novel	[36,37]
	**R257C**	May reduce tobacco-related incidence, however it is uncertain	[35,38]
	V323L	Higher percentage in small cell carcinoma	[37]
	F453Y	Novel	[36]
	R494C	Novel	[36]
CYP2B6	Q21L	Novel	[39]
	R22C	No data on influence	[2,35,40,41,42]
	T26S	-	-
	D28G	-	-
	R29S	-	-
	M46V	Novel	[39]
	G99E	Novel	[39]
	**K139E**	Completely abolished protein expression	[39,42]
	R140Q	Novel	[39]
	P167A	Novel	[35]
	**Q172H**	19.9% frequency in Japanese population, increased V_(max)_ in nonlinear pharmacokinetics	[26,35,40,41,42]
	S259R	Novel	[40,41]
	K262R		[40,41]
	N289K	-	-
	T306S	-	-
	I328T	Novel	[2]
	**I391N**	Leads to undetectable enzyme activity	[39]
	**R487C**	Significantly reduces CYP2B6 protein expression and S-mephenytoin N-demethylase activity, associated with the lowest enzyme activity in females	[35,40,41,42]
CYP2C8	**R139K**	Defective in metabolism of paclitaxel (15% turnover) and arachidonic acid	[2,43,44,45,46,47]
	E154D	No influence from the literature	[48]
	**G171S**	No effect on affinity or enzymatic activity with paclitaxel as substrate; decreases affinity for amodiaquine; reduces enzymatic activity with amodiaquine as substrate; decreases intrinsic clearance of amodiaquine	[46]
	**R186G**	Increases affinity for paclitaxel; reduces enzymatic activity with paclitaxel as substrate; decreases intrinsic clearance of paclitaxel; reduces enzymatic activity with amodiaquine as substrate; decreases intrinsic clearance of amodiaquine	[46]
	N193K	No influence from the literature	[48]
	**I223M**	Reduces enzymatic activity with paclitaxel as substrate; decreases intrinsic clearance of paclitaxel; reduces enzymatic activity with amodiaquine as substrate; decreases intrinsic clearance of amodiaquine	[46]
	**A238P**	Reduces enzymatic activity with paclitaxel as substrate; decreases intrinsic clearance of paclitaxel	[46]
	I244V	-	-
	**K247R**	Increases enzymatic activity with paclitaxel as substrate; reduces enzymatic activity with amodiaquine as substrate; decreases intrinsic clearance of amodiaquine	[46]
	K249R	No influence from the literature	[48]
	**I264M**	Activity towards paclitaxel lower, but not significantly	[44,46,49,50]
	**I269F**	Lower paclitaxel metabolism	[43,44,46]
	**K383N**	Reduces enzymatic activity with paclitaxel as substrate; reduces enzymatic activity with amodiaquine as substrate; decreases intrinsic clearance of amodiaquine	[46]
	**L390S**	In a single subject, coexisted with another polymorphism which caused lower paclitaxel metabolism	[44]
	**K399R**	Defective in paclitaxel and arachidonic acid metabolism	[2,43,44,45,46,50]
	H411L	Novel	[48,51]
	**V461*del***	Increases enzymatic activity with paclitaxel as substrate; reduces enzymatic activity with amodiaquine as substrate; decreases intrinsic clearance of amodiaquine	[46]
CYP2C9	L19I	-	-
	**R125L**	Patients with this variant require a lower warfarin dosage	[52]
	R144C	No correlation to phenytoin, tolbutamide, torasemide and diclofenac metabolism	[53,54,55,56]
	R150H	Novel	[2]
	**N204H**	Restricted binding of the coumarine, resulting in lower metabolism	[57]
	H251R	Novel	[2]
	E272G	-	-
	R335W	Novel	[2,58]
	Y358C	No correlation to phenytoin, tolbutamide, torasemide and diclofenac metabolism	[48,56,59]
	I359L	No correlation to phenytoin, tolbutamide, torasemide and diclofenac metabolism	[55,56]
	**I359T**	Expected to change enzyme activity through ligand binding	[60]
	**D360E**	Lower affinity for warfarin, diclofenac and lauric acid	[2,61]
	L413P	Novel	[2]
	G417D	-	[48,56,59]
	**I434F**	Decrease enzymatic activity in both in vitro and in vivo	[62]
	P489S	-	-
CYP2C19	L17P	Novel, due to its’ proximity to N-terminus probably do not alter enzyme activity	[63]
	I19L	Novel, due to its’ proximity to N-terminus probably do not alter enzyme activity	[63]
	S51G	Novel	[64]
	M74T	Novel	[2]
	**E92D**	Responsible for lower mephenytoin metabolism	[65]
	**W120R**	Reduction in the metabolism of tolbutamide	[66]
	E122A	-	-
	**R132Q**	Responsible for lower mephenytoin metabolism	[65]
	R144H	Novel	[2,63]
	R150H	Novel	[63]
	A161P	Novel	[64]
	F168L	Novel	[2]
	**P227L**	Reduction in catalytic activity	[63]
	R329H	Novel	[64]
	R410C	Novel	[63]
	**R442C**	Location close to the heme region, may result in a decrease in catalytic activity	[67]
CYP2C18	T385M	Novel	[49,68]
CYP2D6	V11M	Novel	[69]
	R25Q	Novel	[69]
	R26H	Novel, no impact found	[2,70]
	R28C	-	-
	**P34S**	Reduction in sparteine metabolism	[2,69,71]
	**G42R**	Found in poor metabolisers, probable reduction in enzyme function	[72]
	A85V	-	-
	L91M	Novel	[2]
	H94R	Novel	[2]
	V104A	-	-
	**T107I**	Possible contribution to diminished debrisoquine hydroxylase activity in African Bantu populations	[2,73]
	F120I	Novel	[2]
	**L142S**	Reduction in catalytic activity of the enzyme	[69]
	K147R	Novel, impact on metabolic activity not found	[69]
	E155K	Novel, impact on metabolic activity not found	[2,70]
	C161S	Novel	[69]
	F164L	-	-
	F164L	Novel	[69]
	**G169R**	Reduction in metabolic activity	[74]
	**G212E**	Premature termination of translation	[75]
	**E215K**	>90% decrease in catalytic activity	[69]
	F219S	Novel	[69]
	A237S	Novel	[2]
	**T249P**	>90% decrease of monooxygenase activity towards dextromethorphan	[60]
	**K281*del***	Decreased metabolism of bufuralol and sparteine	[76]
	**R296C**	Significantly reduces monooxygenase activity toward anandamide	[2,53,60,69,77,78]
	I297L	-	-
	**H324P**	Decreases sparteine metabolism	[79]
	V327M	Novel	[69]
	D336N	Novel	[69]
	D337G	Novel	[69]
	V342M	Novel	[69]
	R343G	-	-
	R344Q	Novel	[69]
	I369T	-	-
	E410K	-	-
	E418K	Novel	[2]
	**R440C**	>90% decrease in catalytic activity	[69]
	F457L	Novel	[69]
	H463D	Novel	[69]
	P469A	Novel	[2]
	H478Y	Novel	[2]
	**S486T**	Associated with lower sparteine metabolism	[2,53,69,70,77,78]
	R497C	Novel	[69]
CYP2E1	**R76H**	Causes 37% of the protein expression and 36% of the catalytic activity compared with the wild-type	[80]
	V179I	No significant difference in pharmacokinetics for chlorzoxazone hydroxylation	[2,81]
	N219D	-	-
	S366C	-	-
	V389I	No significant difference in activity	[80]
	H457L	Novel	[2]
CYP3A4	L15P	Novel, not associated with any change in activity	[82]
	G56D	No change in activity	[83]
	**I118V**	The variant can be linked to 60% less of the main metabolites, however probable new metabolites were observed	[84,85]
	R130Q	No detectable expression found	[83]
	R162Q	No function detected	[82,86]
	V170I	No change in activity	[83]
	D174H	No change in activity	[2,82,83]
	T185S	Novel	[2,84]
	**F189S**	Lower metabolic turnover numbers in *Escherichia coli* for testosterone and chlorpyrifos	[86]
	**P218R**	Suggested reduction in enzyme activity	[85]
	**S222P**	lowered intrinsic clearance for nifedipine	[87]
	S252A	Influence from the literature is unclear	[88]
	**L293P**	Higher metabolic turnover numbers in *Escherichia coli* for testosterone and chlorpyrifos	[2,86]
	**I301T**	May be associated with increased metabolism of 1,25-dihydroxyvitamin D, leading to vitamin-D deficiency rickets infections	[89]
	**T363M**	Lower expression	[83]
	**L373F**	Altered testosterone hydroxylase metabolite profile	[82,83]
	**P416L**	No detectable expression found	[83]
	**I431T**	The connection between the variant and the literature is unclear	[90,91]
	**M445T**	Metabolic turnover numbers in *Escherichia coli* for testosterone and chlorpyrifos not significantly different from the wild variant	[84,85]
	**P467S**	Metabolic turnover numbers in *Escherichia coli* for testosterone and chlorpyrifos not significantly different from the wild variant	[86]
CYP3A5	**R28C**	42–64% lower V_(max)_ for nifedipine oxidation than CYP3A5*1	[91]
	H30Y	Novel	[2]
	Q200R	Novel	[92]
	D277E	Novel	[2]
	**A337T**	42–64% lower V_(max)_ for nifedipine oxidation than CYP3A5*1	[2,91]
	T398N	Novel	[2,92]
	**F446S**	>95% decrease in the intrinsic clearance for both 6β-hydroxytestosterone and nifedipine oxidation	[90]
CYP7A1	F100S	Novel	[93]
	N233S	Novel	[93]
	D347S	The influence is unclear in the literature	[35,94]

**Figure 1:**
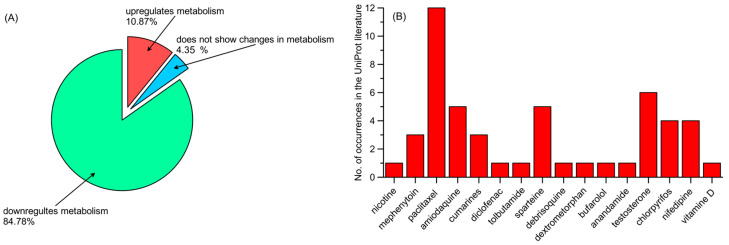
(**A**) Influence of mutations of confirmed relevance on xenobiotic metabolism, and (**B**) xenobiotics metabolized by mutations of confirmed relevance (number of occurrences).

The information provided by the references varies. Some mutations in the enzymes were described in the references solely by their novelty to the scientific world. In these cases, the word “novel” was used. This is frequently the case in reference to CYP1A1. The references may also contain information in accordance with population studies, describing the frequency of the mutation in the overall population or local populations. The data of most interest for us considered the implication of mutations toward the pharmacokinetics of xenobiotics or other evidence of variation-altered enzymatic activities. Table 2 shows the percentage of such relevant variations for each enzyme, with the highest percentage of relevance occurring in CYP3A4 (60%), followed by CYP2C8 (59%), with all other enzymes having less than 50% relevant mutations. We considered both types of xenobiotics being subjected to pharmacokinetics, as well as the direction of metabolism regulation. The metabolism regulation might have been described in the literature with various results. Some of the literature provided results as computable parameters, such as maximum speed (V_(max)_) or clearance [91], whereas others gave us information on protein expression [84], while in other papers, liver activity [25] or clinical outcomes were measured. The results of the analysis of data for these selective mutations are shown in Figure 1. Figure 1A shows a significant overrepresentation of mutations downregulating metabolism compared with other groups (84.78% compared with 10.87% upregulating and 4.348% with changes in metabolism) in the group with confirmed relevance. Figure 1B shows that the mutations with confirmed relevance have the most frequent influence on paclitaxel metabolism (12 occurrences), followed by testosterone (five occurrences), amodiaquine and tolbutamide (both five occurrences).

### 2.2. The CYP3A4 Variations in the Context of Clinical and In Vitro Data

Table 2 presents the percentage of so-called relevant mutations in enzymes that have been identified as clinically significant according to the corresponding references. CYP3A4 exhibits the highest fraction of polymorphic mutations with reported clinical relevance, accounting for 60%. In the current subsection, we present several additional examples of experimental in vitro studies that demonstrate the impact of these CYP3A4 mutations on the metabolic parameters of various xenobiotics. The data shown and discussed here may be treated as a comparative example of how the same mutations translate into pharmacokinetic parameters for different xenobiotics. In relation to the data from Table 1, the data in Table 3 also serve a supplementary function.

Population-level data generally indicate a significantly higher frequency of wild-type enzymes and mutations that do not affect protein structure or sequence across Caucasian, Asian, Native American, and African populations [95,96,97,98,99,100]. For example, 96.86% of such cases were reported in the Chinese Han population [95]. However, some mutations show much higher frequencies in certain populations—for instance, CYP3A4*1B occurs in 76–77% of the African population compared with 3% in the European and 0% in the Asian populations [100,101].

The impact of gender on allele and mutation frequency remains unknown. However, differences in metabolic parameters between males and females have been observed in certain cases. It is generally believed that CYP3A4 exhibits higher metabolic activity in females [101]. Indeed, there are examples of CYP3A4-metabolized xenobiotics showing higher clearance rates in females [102], such as midazolam [103] and triazolam [104]. This may suggest that some mutations contribute to these differences, although this hypothesis has not yet been studied directly.

It is important to note, however, that such cases represent a minority of xenobiotics. For most substances, no significant gender-based differences in metabolism have been observed [102], which diminishes the likelihood that differences in mutation prevalence between sexes significantly influence metabolic outcomes. No data were found regarding the impact of age on these variations.

Given the aforementioned gaps in available data, pharmacokinetic parameters observed in in vitro studies often remain the primary source of information for assessing the impact of specific mutations. The relevant data extracted from the literature are presented in Table 3.

**Table 3:** Metabolic effects of CYP3A4 mutations on metabolic activity toward xenobiotics in vitro. The parameters (V_(max)_—the maximum rate of metabolic reactions; K_M_—Michaelis–Menten constant; Cl—intrinsic clearance) were determined with respect to the wild type; ↓ the parameter is lower than in wild type, ↑ the parameter is higher than in wild type, ↕ the parameter change is not statistically significant. * denoted standardized star allele nomenclature.

Allele (*)	Mutation Code	Relevance (with Respect to Table 1)	Lidocaine Fang et al. [7]	Loperamide Lin et al. [105]	Imatinib Chen et al. [106]	Sildenafil Tang et al. [4]	Abemaciclib Xu et al. [107]
1	Wild type	+	model	model	Model	model	model
2	S222P	+	↓ V_(max)_ ↑ K_M_ ↓ Cl	↓ V_(max)_ ↑ K_M_ ↓ Cl	↓ V_(max)_ ↕ K_M_ ↓Cl	↑ V_(max)_ ↑ K_M_	No data
3	M445T	+	↑ V_(max)_ ↑ K_M_ ↕ Cl	↕ V_(max)_ ↑ K_M_ ↓ Cl	↓ V_(max)_ ↕ K_M_ ↕ Cl	↑ V_(max)_ ↕ K_M_	↑ V_(max)_ ↑ K_M_ ↑Cl
4	I118V	+	No effect	↓ V_(max)_ ↑ K_M_ ↓ Cl	↓ V_(max)_ ↑ K_M_ ↓ Cl	↑ V_(max)_ ↕ K_M_	No data
5	P218R	+	↓ V_(max)_ ↑ K_M_ ↓ Cl	↕ V_(max)_ ↑ K_M_ ↓ Cl	↓ V_(max)_ ↑ K_M_ ↓ Cl	↓ V_(max)_ ↑ K_M_	No data
7	G56D	-	Not mentioned	↑ V_(max)_ ↑ K_M_ ↓ Cl	↓ V_(max)_ ↕ K_M_ ↓ Cl	No data	No data
8	R130Q	-	Not mentioned	↑ V_(max)_ ↑ K_M_ ↓ Cl	↓ V_(max)_ ↑ K_M_ ↓ Cl	No data	No data
9	V170I	-	↕ V_(max)_ ↑ K_M_ ↓ Cl	↓ V_(max)_ ↕ K_M_ ↓ Cl	↓ V_(max)_ ↑ K_M_ ↓ Cl	↑ V_(max)_ ↑ K_M_	No data
10	D174H	-	No effect	↓ V_(max)_ ↕ K_M_ ↓ Cl	↓ V_(max)_ ↑ K_M_ ↓ Cl	↑ V_(max)_ ↕ K_M_	No data
11	T363M	+	↑ Cl	↑ V_(max)_ ↑ K_M_ ↓ Cl	↓ V_(max)_ ↕ K_M_ ↓ Cl	↑ V_(max)_ ↑ K_M_	No data
12	L373F	+	Not mentioned	↓ V_(max)_ ↑ K_M_ ↓ Cl	↓ V_(max)_ ↑ K_M_ ↓ Cl	No data	No data
13	P416L	+	Not mentioned	↓ V_(max)_ ↑ K_M_ ↓Cl	↓ V_(max)_ ↑ K_M_ ↓ Cl	No data	No data
14	L15P	-	↑ Cl	↑ V_(max)_ ↑ K_M_ ↓Cl	↑ V_(max)_ ↑ K_M_ ↑ Cl	↑ V_(max)_ ↓ K_M_	No data
15	R126Q	-	↑ Cl	↓ V_(max)_ ↑ K_M_ ↓Cl	↕ V_(max)_ ↑ K_M_ ↓ Cl	↑ V_(max)_ ↕ K_M_	↑ V_(max)_ ↑ K_M_ ↑ Cl
16	T185S	-	↓ V_(max)_ ↑ K_M_ ↓ Cl	↑ V_(max)_ ↑ K_M_ ↓ Cl	↓ V_(max)_ ↑ K_M_ ↓ Cl	No data	No data
17	F189S	+	Extremely lower activity	No data	↓ V_(max)_ ↑ K_M_ ↓ Cl	No data	No data
18	L293P	+	↑ V_(max)_ ↑ Cl	↑ V_(max)_ ↑ K_M_ ↓ Cl	↓ V_(max)_ ↑ K_M_ ↓ Cl	No data	↑ V_(max)_ ↑ K_M_ ↑ Cl
19	P467S	+	↑ Cl	↓ V_(max)_ ↕ K_M_ ↓ Cl	↓ V_(max)_ ↑ K_M_ ↓ Cl	↑ V_(max)_ ↕ K_M_	No data

These data show that the effects of various enzyme variants—including those classified as relevant based on the data in Table 1—can differ depending on the type of xenobiotic being metabolized. Only a few variants exhibit a consistent influence on pharmacokinetics. For instance, the P218R variant demonstrates a stable effect, decreasing both V_(max)_ (although in one case the reduction was not statistically significant) and clearance (Cl), while increasing the K_M_. A similarly consistent trend, allowing for minor statistically insignificant deviations, is also observed for the I118V, L373F, and P416L variants—although in the latter two cases, the pool of tested xenobiotics is rather limited. In contrast, most of the analyzed variants show inconsistent effects across different compounds. Some variants, such as L15P, simultaneously increase both V_(max)_ and K_M_, which is considered an atypical outcome. Several variants significantly affect intrinsic clearance—for example, S222P, P218R, V170I, and T185S—all of which lead to a reduction in this parameter.

The comparison indicates that, although certain pharmacokinetic tendencies can be predicted, the direction and magnitude of a variant’s influence must be determined separately for each xenobiotic.

### 2.3. Analysis of Existing Variants in the Context of Enzyme Structures

The mutations listed in Table 1, corresponding to different variants of the considered CYP450 enzymes, were analyzed in the context of the structural properties of the enzymes and the locations of these mutations. This analysis is qualitative in nature, attempting to link the location and characteristics of a given mutation with its possible consequences for the enzyme’s mechanism of action. Such an approach is largely speculative due to the lack of data that clearly confirms the impact of every given variant. However, the results of this type of analysis may be of great importance in identifying potential mechanisms through which polymorphisms affect the function of CYP450 enzymes, as well as exploring potential research directions focused on specific enzyme/polymorph cases. Moreover, in the next section, we will explore the specific polymorphs of a given enzyme for which its importance has been reported in the literature.

The analyses presented in this section are based on the structures of the respective proteins deposited in the AlphaFold database (alphafold.ebi.ac.uk). This choice, alternative to selecting structures from the PDB database (www.rcsb.org), was dictated by the need to consider the full structure of the proteins, including fragments that may be absent in experimentally resolved structures (for example, none of the structures in the PDB database contain structural data corresponding to the transmembrane alpha-helix). Additionally, the CYP450 enzyme models generated by AlphaFold are characterized by very high (pLDDT > 90) confidence for structured fragments and high (90 > pLDDT > 70) confidence for fragments that are typically absent from structural data (pLDDT stands for the predicted Local Distance Difference Test, a confidence score used in protein structure prediction). Finally, the present analysis is primarily qualitative, and minor inaccuracies in the structural model do not affect the final conclusions.

**Figure 2:**
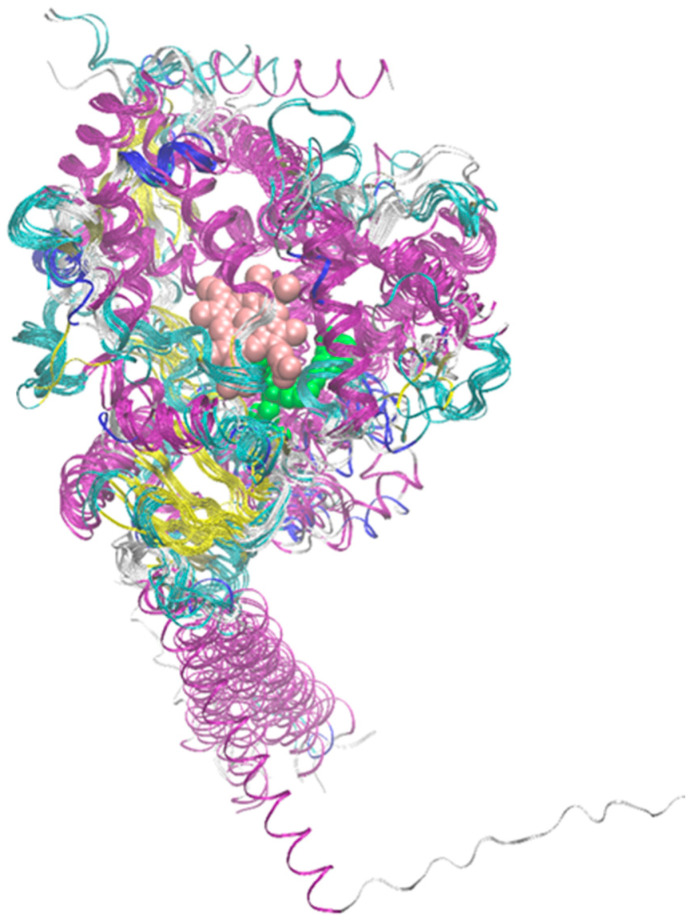
Superposition of the structures of the 15 considered enzymes from the CYP450 family. The color code corresponds to the secondary structure. Additionally, the positions of the heme molecule and an example ligand are marked (van der Waals spheres colored pink and green, respectively). In the latter case, structure PDB:2NNI was used.

It is worth noting at the outset that all 15 enzymes considered here have highly similar secondary structures, despite significant differences in sequence. For example, the average percent identity is 36.3%, with the highest value, recorded for CYP2C19 and CYP2C9, being 91.4%. The corresponding matrix showing percent identity for the entire group of considered enzymes is presented in Figure S2. Meanwhile, the average RMSD (root mean square deviation) value for the superimposed structures shows very limited variability in the range of 0.09–0.26 nm per non-hydrogen atom. Figure S1 presents a sequence comparison performed using the online Omega Clustal program (ebi.ac.uk/jdispatcher/msa/clustalo, accessed on 6 August 2025), while Figure 2 shows the superimposed structures of the 15 examined enzymes with the minimal RMSD value for a single atom of the structure.

Figure S1 (Supporting Information) shows the sequence alignment for all enzymes considered in this study, along with mutation sites specified in Table 1 and described in the appropriate section. A qualitative insight into the locations corresponding to the variants described above shows that they are not confined to single, corresponding regions in the molecule but are scattered across many areas of the sequence. However, several distinct regions can be observed where mutations leading to different variants are overrepresented.

**Figure 3:**
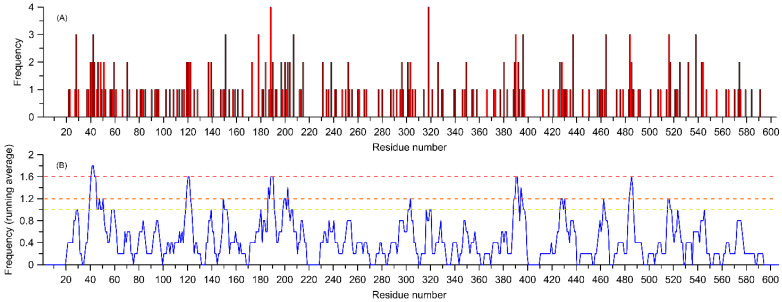
Frequency of occurrence of the variants listed in Table 1 assigned to aligned sequences of 15 considered amino acid residues. The independent variable is the number of the hypothetical amino acid unit (HAU), corresponding to the column number for the overlapping sequences (Figure S1). Panel (**A**) shows the absolute frequencies of variant occurrences, while panel (**B**) displays the running average for the same value calculated for 5 neighboring residues (including sequence gaps). The horizontal lines in panel (**B**) correspond to frequency levels for the amino acids marked with the same colors in Figure 4A.

Figure 3A shows the frequency of variant occurrence relative to a given position in the sequence, while Figure 3B presents the same value as a running average over 5 neighboring residues. In both cases, the independent variable is the number of the hypothetical amino acid unit (HAU), corresponding to the column number for the superimposed sequences (Figure S1).

Such a quantitative comparison shows that, for the 15 analyzed enzymes, the regions most susceptible to the occurrence of biologically relevant variants are the following:

1. The region of the transmembrane helix (HAU = 40–50);
2. The loop closing the entrance to the ligand binding cavity (HAU ≈ 120);
3. The loop in contact with the heme molecule and involved in the catalytic reaction with CYP450 protein partners (HAU ≈ 516);
4. Other regions of the CYP450 molecule that interact with the protein partner but are not part of region (C) (HAU ≈ 150, 303, 318, 390, 485, 523);
5. Regions that may interact with larger ligands and are located on alpha-helices adjacent to the binding pocket (HAU ≈ 205 and 320).

The remaining regions, not included in those listed above, are mainly located on an alpha-helix (HAU ≈ 190) and the adjacent loop fragment (HAU ≈ 200).

**Figure 4:**
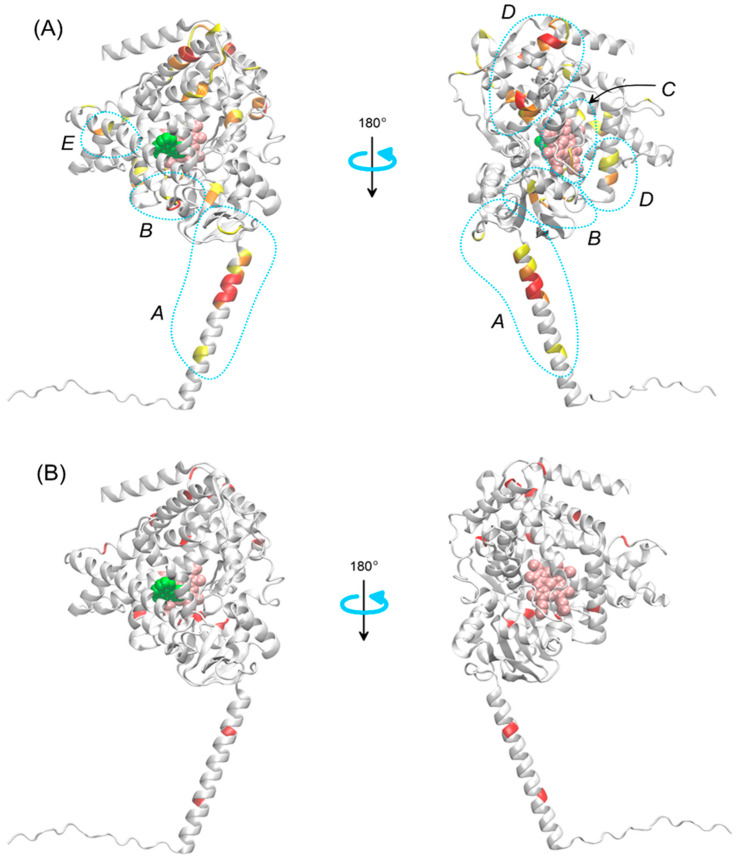
(**A**) Regions of the CYP450 enzyme family most susceptible to polymorphic changes with experimentally confirmed significance (see Table 1). The regions are denoted by letters A–E and explained in the text. The illustration is based on the structure of CYP1B1. The color code corresponds to the weighted average values from Figure 3B, with three colors representing three value levels: above 1 (yellow), above 1.2 (orange), and above 1.6 (red). Regions where polymorphic changes exert an effect through different elements of the enzyme’s mechanism of action are marked (blue dotted lines). (**B**) Same as in panel (**A**), but with polymorphic change sites marked in red, corresponding to the same HAU value and occurring with a frequency of 3 or more (out of a set of 15 enzymes). Other details as in Figure 2.

Regarding the frequency of variant occurrence at a specific site in the sequence, out of 277 variants, nearly half occur only once, at a specific site in a single enzyme. There are 52 variants that involve the same site in two different enzymes, and 12 variants that involve the same site in three different enzymes. Finally, there are two variants with a site recurring in four different enzymes. This comparison does not consider the specific types of mutations, but only their locations. Figure 4B shows the locations of variants that most frequently (three or four times) appear among the 15 considered enzymes, mapped onto the structure of CYP1B1.

The location of the most common polymorphs, with a frequency of three or more among the analyzed group of enzymes (Figure 4B), usually corresponds to the regions with the highest concentration of variants (cf. Panels (A) and (B) in Figure 3 and Figure 4A), and thus, this will not be discussed separately. However, it is worth presenting the specific enzymes and sites corresponding to the most frequently recurring variants: HAU = 28 for CYP3A4, CYP2D6 and CYP1B1 (L, V and S are exchanged, respectively); HAU = 42 for CYP2D6, CYP2B6 and CYP2A13 (R is exchanged in all cases); HAU = 151 for CYP3A4, CYP2C9 and CYP2A6 (R is exchanged in all cases); HAU = 178 for CYP2C9, CYP2C19 and CYP2A6 (R is exchanged in all cases); HAU = 188 for CYP2C8, CYP2A6, CYP2A13 and CYP1B1 (E, D, D and A are exchanged, respectively); HAU = 207 for CYP2C8, CYP2B6 and CYP1B1 (G, Q and V are exchanged, respectively); HAU = 318 for CYP2B6, CYP1B1, CYP1A1 and CYP1A2 (K, R, R and R are exchanged, respectively); HAU = 390 for CYP3A5, CYP2D6 and CYP2C19 (A, D and R are exchanged, respectively); HAU = 396 for CYP2D6, CYP2C9 and CYP1B1 (R is exchanged in all cases); HAU = 437 for CYP3A4, CYP1B1 and CYP1A2 (L, I and I are exchanged, respectively); HAU = 464 for CYP2C8, CYP2B6 and CYP1B1 (L, I and N are exchanged, respectively); HAU = 484 for CYP2D6, CYP2C19 and CYP1B1 (E, R and A are exchanged, respectively); HAU = 516 for CYP2D6, CYP2C19 and CYP1A2 (R is exchanged in all cases) and HAU = 538 for CYP2D6, CYP2E1 and CYP1A1 (H, H and R are exchanged, respectively). Even based on this sample, it can be stated that mutations leading to the substitution of arginine are overrepresented. This issue will be explored based on the full data set.

Division of the most common polymorphisms based on their location within the enzyme molecule reflects potential mechanisms through which they may affect the physiological functions of the protein. Below, we discuss the potential impact of variants located in each of the aforementioned regions.

#### 2.3.1. Transmembrane Helix

All enzymes from the CYP450 family are transmembrane proteins, and their embedding in the membrane significantly influences their function [108,109,110], particularly in terms of binding to redox partners [111] and the migration of substrates and products through the membrane environment [112]. Membrane-helix interactions are determined, among others, by the amino acid sequences that form the helix and affect the stability of the protein within the membrane, which in turn influences interactions with redox partners [113] and catalytic activity [114]. It is, therefore, not surprising that mutations within the transmembrane helix (region A in Figure 4A,B), seemingly distant from the catalytic site, can be significant for the functioning of CYP450 enzymes. Recent studies based on molecular dynamics simulations indicate that single mutations in CYP19 [115] and CYP17A1 [116] affect the orientation of the protein in the membrane, which may, in turn, impact the course and efficiency of the catalytic reaction.

#### 2.3.2. Substrate/Product Entry/Exit Channels

As shown by many studies (see, for example, the review article [117] on the entire family or articles on specific enzymes [118,119,120,121]), the paths of ligand entry and exit from the binding pocket can differ even for the same ligand and may also depend on the characteristics of the ligand itself and on dynamic conformational changes occurring in the enzyme molecule. The typical locations of ligand migration paths to/from the binding site are located near region B, marked in Figure 4A,B. Polymorphic changes in this region are quite common and can certainly influence the enzyme’s action by altering the free energy profile associated with ligand migration along a given channel.

#### 2.3.3. Heme Pocket Region

The activity of redox partner proteins of CYP450 enzymes occurs through the formation of a protein–protein complex, enabling a reaction in which the heme molecule within CYP450 is directly involved. Proteins that form complexes with CYP450 and participate in the reaction are called redox partners [122]. In reactions involving redox partners, CYP450 reductase usually provides the essential first electron, while the second electron may be delivered either directly by the reductase or via cytochrome b5 [123]. Although CYP450-redox partner protein complexes are usually not experimentally resolved (with exceptions, e.g., ref. [124], PDB:1BVY), knowledge about the course of the reaction and alternative structural characterization methods (especially molecular modeling) allow for the identification of the association interface of the appropriate complexes, as well as the key interactions responsible for the stability of protein–protein binding. Particularly important for the redox reaction is the loop in proximity to the heme molecule. Although the sequence in this region is usually conserved (GKR triad in 12 out of 15 enzymes), in three of them the arginine undergoes mutation, which may affect the reaction process occurring in the immediate vicinity [122].

#### 2.3.4. P450/Redox Partner Contact Interface

**Figure 5:**
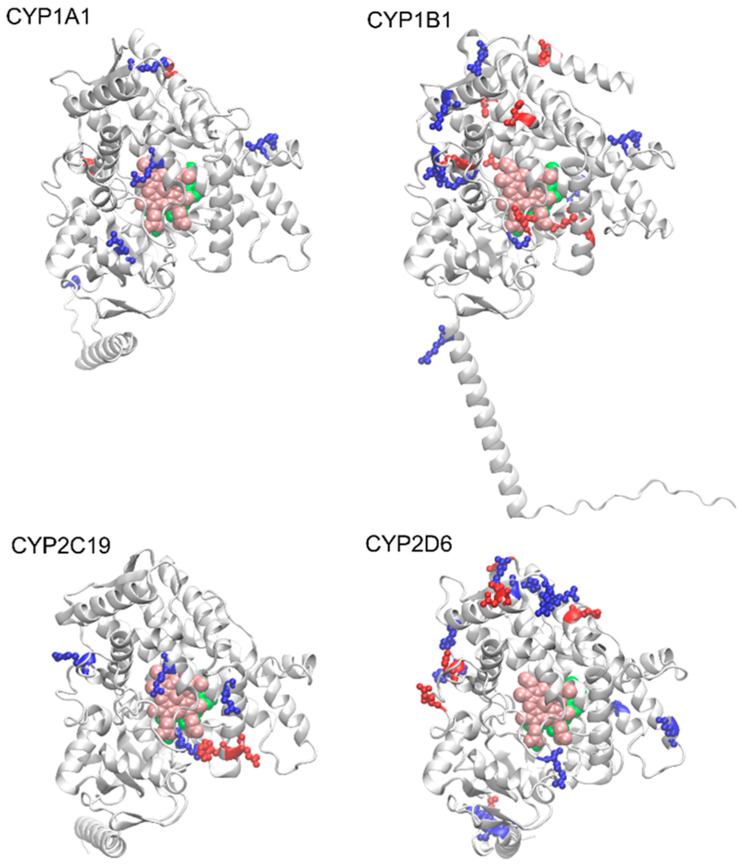
Structures of the enzymes CYP1A1, CYP1B1, CYP2C19, and CYP2D6, showing the locations of variants where a mutation leads to a change in the charge of a given residue. The color code corresponds to the direction of the change: red indicates an increase in charge value, and blue indicates a decrease in charge value. Other details as in Figure 2.

The above point can be extended to a more general case of contact between CYP450 and the redox partner, not limited to the reaction site. Numerous reports on the structure of complexes between CYP450 family proteins and redox partners [123,124,125,126,127,128,129,130] suggest a relatively large surface area involved in protein–protein contact; the relevant interface approximately overlaps with regions C and D in Figure 4A. Moreover, protein–protein binding occurs largely through complementary electrostatic interactions [111], including salt bridges, as demonstrated not only by structural studies but also by experiments involving ionic strength modifications [108,131]. Mutations involving the potential contact interface between proteins often include substitutions of charged residues with uncharged ones (and vice versa), leading to significant changes in the electrostatic potential near the mutation and alterations in the strength of protein binding. Figure 5 illustrates mutations in four example enzymes that lead to changes in electric charge at the CYP450–redox partner contact interface. As a further example of the importance of such mutations, it is worth noting that residues D337 and R440 of the CYP2D6 enzyme, identified [126] as key for contact with cytochrome P450 reductase during the relevant catalytic reaction, are located in regions particularly prone to polymorphic mutations (the same residues are also among the set of considered mutations; see Table 1).

#### 2.3.5. Helices near the Binding Pocket

Although the most mutation-prone sites are not located in the immediate vicinity of the ligand or the ligand migration pathway to/from the binding pocket, they are close enough that residue substitutions in this region can affect the stability of the binding pocket, thereby impacting the reaction itself, as well as the ligand migration processes.

Additionally, it is worth noting the potential impact of individual polymorphs that do not show more than a single occurrence within the entire group, yet constitute nearly 50% of all variants listed in Table 1. Although we did not conduct an analysis for these cases, it is worth noting that a large portion of them are located in or near the regions described above, as well as in areas enabling interaction with elements crucial to the catalytic reaction, such as the ligand, the heme molecule, or catalytic residues. Changes in the type/intensity of interactions due to mutations may also affect, among others, the stability of the ligand molecule in the binding pocket, migration of substrates/products to/from the binding pocket, the course of the reaction, and the enzyme’s interaction with redox partners.

**Figure 6:**
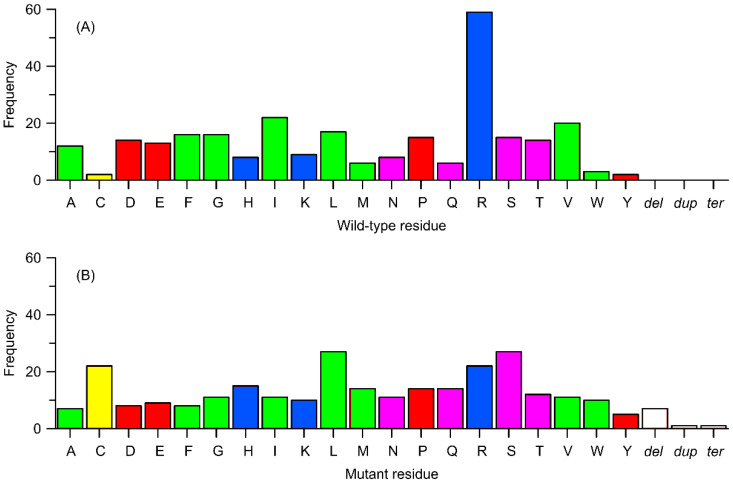
Frequency of occurrence of variants from Table 1 in relation to the type of change (substitution to a different amino acid, deletion, termination, or duplication). (**A**) Replaced residues corresponding to wild-type. (**B**) Residues replacing the wild-type ones. Amino acids are color-coded as follows: yellow—cysteine (Cys), green—nonpolar residues, pink—polar uncharged residues, blue—positively charged residues, and red—negatively charged residues. Other details: dup = duplication of a sequence, del = deletion of a sequence, and ter = a termination (stop) codon.

In the context of frequencies that do not involve the exchange of specific residue positions but rather the substitution of a given type of residue, the corresponding analysis (Figure 6) shows that the most frequently exchanged residue type is arginine (21% of all cases), followed by isoleucine and valine (8% and 7% of cases, respectively). The remaining residues are substituted in 1–6% of cases, with cysteine, tyrosine, and tryptophan being the least prone to substitution (1%). It is worth noting the significant value (5.5%) associated with proline and other charged residues (3–5%) besides arginine. The substantial contribution of charged residues is consistent with a possible mechanism of action of the polymorphism discussed in sections C and D above. Furthermore, the results explain the non-negligible impact of polymorphisms involving arginine (and other charged residues), considering that it is often involved in conservative, ionic interactions, and substituting it with neutral residues can lead to the loss of critical interactions.

In this context, it is important to note that as many as 59 mutations (21% of all considered variants) lead to a decrease (by 1 or more *e*^−^) in the protein’s electric charge, whereas the corresponding values leading to an increase of 1 *e*^−^ or more are 41 and 15%, respectively. In addition to charged residues, it is noteworthy that proline, i.e., an amino acid critical for structural integrity, when mutated, can influence local changes in protein structure and, indirectly, the reactions involving the enzyme.

As for the type of residue replacing the native one in a given polymorphism, there is no such clear disparity. The most common residues present after substitution are serine and leucine (just under 10%), while the least represented is tyrosine (2%). Considering the relative frequencies, the largest decrease in population between the native and mutated residue can be noted for arginine (13%), while the largest increase is observed for cysteine (7%).

### 2.4. Variants of Confirmed Relevance

Unlike the previous section, where all existing variants of enzymes were discussed, in this section, we will focus only on the subgroup of those variants whose relevance has been confirmed in various types of experimental studies. Details are provided in Table 1, and corresponding variants are marked with bolded font. Additionally, in this subsection, the enzyme CYP1B1 was excluded from discussion. The reason for that was given in one of the previous sections. Apart from that, from the pool of enzymes discussed in this subsection, CYP7A1 and CYP2C18 were also excluded due to a lack of confirmation of the significance of any of the variants listed in Table 1.

From the total pool of all variants collected in Table 1, only 59 have experimentally or clinically confirmed significance, translating to physiological function. This constitutes approximately 28% of the total, excluding the case of CYP1B1, which is not discussed here.

The distribution of variants with confirmed significance in the enzyme structures is, similarly to the case of all variants discussed above, very diverse and includes different parts of the enzyme corresponding to regions highlighted in Figure 4. Additionally, despite the lack of specific reports regarding the structural causes of the effects exerted by a given mutation, in this case as well, the potential mechanisms broadly described in the previous subsection are relevant. In particular, the following were noted:

1. Two mutations in region A (transmembrane helix) occurring in CYP1A2 and CYP3A5;
2. Four mutations in region B (substrate/product entry/exit channels) for CYP3A4, CYP2D6, CYP2C8, and CYP2E1;
3. Frequent (>8) mutations in region C (heme pocket) observed in CYP2B6, CYP2C8, CYP2C9, CYP2C19, CYP2D6, CYP1A1, CYP3A4, and CYP3A5;
4. Equally frequent mutations, in terms of number of proteins and more frequent in terms of number of mutations, in region D (P450/redox partner contact interface) for CYP2B6, CYP2C9, CYP2C19, CYP2D6, CYP1A1, CYP3A4, and CYP3A5; partly, this group overlaps with the group from point (3);
5. Numerous mutations in region E (helices and other regions near the ligand binding pocket) for CYP2C6, CYP2C9, CYP2D6, CYP2A6, and CYP3A4;
6. Mutations in other regions of the protein, not directly associated with intuitively key structural elements for the enzyme’s mechanism of action but potentially influencing its dynamic structure, for example, mutations in short loops connecting adjacent helices or substituting amino acids with drastically different properties; examples correspond to enzymes CYP2A13, CYP2B6, CYP2C8, CYP2C19, CYP2D6, CYP1A2, and CYP3A4.

In the context of mutations leading to a change in the overall charge of the protein, such cases are even more frequent in the considered subset of mutations than in the entire data pool. Such mutations account for as many as 23 out of 59 cases (approximately 39%), of which eight are cases of increased charge and the rest decreased charge. Except for one case where the charge change was as high as +2*e*^−^, the remaining cases corresponded to a unit change. This type of change often, though not always, concentrates in the region of the interface with the redox partner and/or in the heme or ligand binding pocket (e.g., R431W in CYP1A1, R125L in CYP2C9, W120R, R132Q, and R442C in CYP2C19, as well as K281*del* and R440C in CYP2D6). These cases, due to the importance of electrostatic interactions for the binding of CYP enzymes with their redox partners, indicate a significant potential impact on the mechanism of enzyme action.

**Figure 7:**
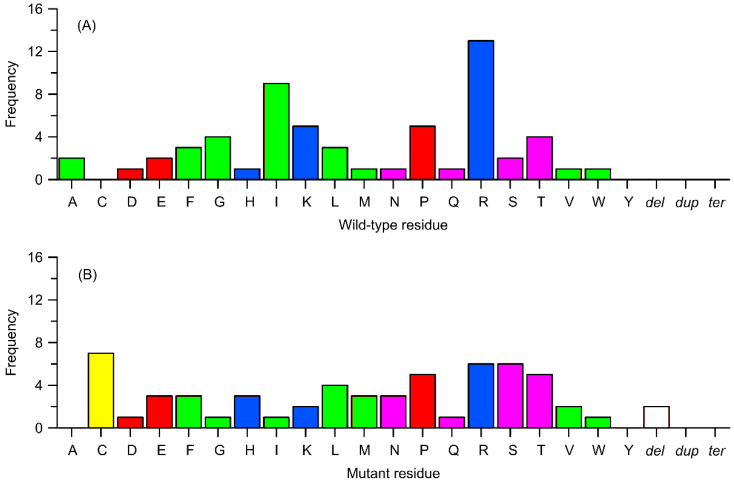
Frequency of occurrence of subset of variants from Table 1, identified as biologically relevant, in relation to the type of change (substitution to a different amino acid, deletion, termination, or duplication). (**A**) Replaced residues corresponding to wild-type ones. (**B**) Residues replacing the wild-type ones. Other details as in Figure 6.

Figure 7 presents data analogous to those illustrated in Figure 6, i.e., the frequency of polymorphic changes concerning the replaced amino acid and the amino acid substituting the replaced residue; in this case, the focus is on the subgroup of mutations with confirmed effects on enzyme activity. Again, the most frequently replaced residue (21%) is arginine, followed by isoleucine (15%), lysine (8%), and proline (8%). On the other hand, the residues most frequently appearing as a result of mutations are cysteine (11.5%), followed by serine and arginine (10%) and threonine (8%). Once more, a significant overrepresentation of positively charged residues and proline, important from the standpoint of preserving secondary structure, can be observed.

### 2.5. Perspectives of Molecular Modeling-Based Studies

Apart from well-established experimental and clinical studies, future research should employ a combination of advanced molecular modeling techniques to better understand how polymorphisms influence the CYP450 enzymes’ function. For instance, highly efficient coarse-grained molecular dynamics simulations can provide valuable insights into how polymorphic variations (and other mutations) affect the orientation of CYP450 enzymes within the membrane and modulate their interactions with redox partners. Atomistic molecular dynamics simulations (a tool that has already been used numerous times to study the issue of polymorphisms of CYP450 members) are essential for exploring detailed conformational dynamics of the whole proteins and their crucial regions, ligand migration pathways and the stability of both the heme molecule and bound substrates. Furthermore, quantum mechanics-based approaches may enable accurate investigation of the catalytic reaction mechanism and the effects of mutations that alter the associated energy landscape, including the influence of local electrostatics, particularly near the active site or redox partner interface. Integrating these computational methods with experimental data will be critical for unraveling the complex and diverse structural impacts of polymorphisms on CYP450 activity. Importantly, we plan to apply several of these theoretical approaches in our future studies to provide a more comprehensive molecular understanding of CYP450 variants and their pharmacological implications.

## 3. Summary

A qualitative analysis based on examining the location and type of mutations in relation to enzyme structure indicates that there is no single, universal mechanism—common to all CYP450 family enzymes or even to individual members of the family—by which polymorphism may influence their function. However, it is possible to distinguish several distinct structural factors that affect the significance of polymorphisms under physiological conditions. These are, namely, the following:

1. Mutations within the transmembrane helix, which affect the orientation of the CYP450 molecule relative to the membrane and its redox partners;
2. Mutations within the migration channels of ligands (reaction substrates and products) to and from the binding site, which alter the kinetic characteristics of the functioning enzyme;
3. Mutations at the contact interface between the CYP450 molecule and its redox partner, including those located in the immediate vicinity of the catalytic heme group.

Regarding the last point, it is worth noting that the considered polymorphisms are very often (in 36% of cases in relation to all occurring variants and 39% in the case of variants of confirmed biological relevance) associated with a change in the charge of the mutated residue, with arginine being the most frequently substituted residue. This may be highly significant for the course of reactions involving redox partners, given that the interactions responsible for protein association are electrostatic in nature.

The analysis also highlighted some important issues concerning the influence of the natural, polymorphic mutations on the metabolism of xenobiotics. The significant overrepresentation of downregulating mutations shows us that most of them generally improve the effectiveness of drug administration while also increasing the risk of side effects, presumably the opposite for prodrugs. The most frequent influence of the mutations on paclitaxel, being a cytostatic drug, indicates that these mutations may have an influence on drugs with frequent side effects, therefore making the subject of mutations an important issue for further studies and analyses. In vitro studies, considered here in the context of CYP3A4, demonstrate that the influence of a given type of mutation may be non-systematic with respect to different xenobiotics.

## Figures and Tables

**Table 2:** Percentage of relevant mutations in each of the considered CYP450 enzymes.

Enzyme	Relevant Mutations	Mutations Overall	No. of Relevant Mutations	% of Relevant Mutations
CYP1A1	T461N, I462V	14	2	14.3
CYP1A2	F21L, R431W	22	2	9.1
CYP1B1	Not considered			
CYP2A6	R203C	19	1	5.3
CYP2A13	R257C	8	1	12.5
CYP2B6	K139E, Q172H, I391N, R487C	18	4	22.2
CYP2C8	R139K, G171S, R186G, I223M, A238P, K247R, I264M, I269F, K383N, L390S, K399R, V461*del*	17	12	70.5
CYP2C9	R125L, N204H, I359T, D360E, I434F	16	5	31.3
CYP2C19	E92D, W120R, R132Q, P227L, R442C	16	5	31.3
CYP2C18	-	1	0	0.0
CYP2D6	P34S, G42R, T107I, L142S, G169R, G212E, E215K, T249P, K281*del*, R296C, H324P, R440C, S486T	44	13	29.6
CYP2E1	R76H	6	1	16.7
CYP3A4	I118V, F189S, P218R, S222P, L293P, I301T, T363M, L373F, P416L, I431T, M445T, P467S	20	12	60.0
CYP3A5	R28C, A337T, F446S	7	3	42.9
CYP7A1	0	3	0	0.0

## Data Availability

The dataset is available on request from the authors.

## Supplementary Materials

The supporting information can be downloaded at https://www.mdpi.com/article/10.3390/ijms26167797/s1.
