# Supplementary material for: Natural Polymorphic Variants in the CYP450 Superfamily: A Review of Potential Structural Mechanisms and Functional Consequences
Source: Int J Mol Sci. 2025 Aug 12;26(16):7797. doi: 10.3390/ijms26167797 (PMC12386198; doi:10.3390/ijms26167797)
Supplement: Supplementary file 1 [file ijms-26-07797-s001.zip › ijms-3767074-supplementary.pdf]

HAU:	1–60	
CYP7A1	- - - - - - - - - - - - - - - - M M T T S L I W G I A I A A C C C L W L I L G I R R R Q T G E P - - P L E N G L I P Y L	42
CYP3A4	- - - - - - - - - M A L I P D L A M E T W - - - - L L L A V S L V L L Y L Y G T H S H G L F K K L G I P G P T P L P F L	47
CYP3A5	- - - - - - - - - M D L I P N L A V E T W - - - - L L L A V S L V L L Y L Y G T R T H G L F K R L G I P G P T P L P L L	47
CYP2D6	- - - - - - - - - - - - - - - - - M G L E A L V P L A V I V A I F L L L V D L M H R R Q R W A A R Y P P G P L P L P G L	43
CYP2E1	- - - - - - - - - - - - - - - - - - M S A L G V T V A L L V W A A F L L L V S M W R Q V H S S W N L P P G P F P L P I I	42
CYP2C8	- M E P F V V L V L C L S F M L L F S L W R Q S C R R R K L P P G P T P L P I I	39
CYP2C18	- M D P A V A L V L C L S C L F L L S L W R Q S S G R G R L P S G P T P L P I I	39
CYP2C9	- M D S L V V L V L C L S C L L L L S L W R Q S S G R G K L P P G P T P L P V I	39
CYP2C19	- M D P F V V L V L C L S C L L L L S I W R Q S S G R G K L P P G P T P L P V I	39
CYP2B6	- - - - - - - - - - - - - - - - - M E L S V L L - - - F L A L L T G L L L L L V Q R H P N T H D R L P P G P R P L P L L	40
CYP2A6	- - - - - - - - - - - - - - - - - M L A S G M L L V A L L V C L T V M V L M S V W Q Q R K S K G K L P P G P T P L P F I	43
CYP2A13	- - - - - - - - - - - - - - - - - M L A S G L L L V T L L A C L T V M V L M S V W R Q R K S R G K L P P G P T P L P F I	43
CYP1B1	M G T S L S P N D P W P L N P L S I Q Q T T L L L L L S V L A T V H V G Q R L L R Q R R R Q L R S A P P G P F A W P L I	60
CYP1A1	- - - - - - - - - - - M L F P I S M S A T E F L L A S V I F C L V F W V I R A S R P Q V P K G L K N P P G P W G W P L I	49
CYP1A2	- - - - - - - - - M A L S Q S V P F S A T E L L L A S A I F C L V F W V L K G L R P R V P K G L K S P P E P W G W P L L	51

HAU:	61–120	
CYP7A1	G C A L Q F G - A N P L E F L R A N Q R K H G H V F T C K L M G K Y V H F I T N P L S Y H K V L C H G K Y F D W K K - F	100
CYP3A4	G N I L S Y H - K G F C M F D M E C H K K Y G K V W G F Y D G Q Q P V L A I T D P D M I K T V L V K E C Y S V F T N R R	106
CYP3A5	G N V L S Y R - Q G L W K F D T E C Y K K Y G K M W G T Y E G Q L P V L A I T D P D V I R T V L V K E C Y S V F T N R R	106
CYP2D6	G N L L H V D F Q N T P Y C F D Q L R R R F G D V F S L Q L A W T P V V V L N G L A A V R E A L V T H G E - D T A D R P	102
CYP2E1	G N L F Q L E L K N I P K S F T R L A Q R F G P V F T L Y V G S Q R M V V M H G Y K A V K E A L L D Y K D - E F S G R G	101
CYP2C8	G N M L Q I D V K D I C K S F T N F S K V Y G P V F T V Y F G M N P I V V F H G Y E A V K E A L I D N G E - E F S G R G	98
CYP2C18	G N I L Q L D V K D M S K S L T N F S K V Y G P V F T V Y F G L K P I V V L H G Y E A V K E A L I D H G E - E F S G R G	98
CYP2C9	G N I L Q I G I K D I S K S L T N L S K V Y G P V F T L Y F G L K P I V V L H G Y E A V K E A L I D L G E - E F S G R G	98
CYP2C19	G N I L Q I D I K D V S K S L T N L S K I Y G P V F T L Y F G L E R M V V L H G Y E V V K E A L I D L G E - E F S G R G	98
CYP2B6	G N L L Q M D R R G L L K S F L R F R E K Y G D V F T V H L G P R P V V M L C G V E A I R E A L V D K A E - A F S G R G	99
CYP2A6	G N Y L Q L N T E Q M Y N S L M K I S E R Y G P V F T I H L G P R R V V V L C G H D A V R E A L V D Q A E - E F S G R G	102
CYP2A13	G N Y L Q L N T E Q M Y N S L M K I S E R Y G P V F T I H L G P R R V V V L C G H D A V K E A L V D Q A E - E F S G R G	102
CYP1B1	G N A A A V G - Q A A H L S F A R L A R R Y G D V F Q I R L G S C P I V V L N G E R A I H Q A L V Q Q G S - A F A D R P	118
CYP1A1	G H M L T L G - K N P H L A L S R M S Q Q Y G D V L Q I R I G S T P V V V L S G L D T I R Q A L V R Q G D - D F K G R P	107
CYP1A2	G H V L T L G - K N P H L A L S R M S Q R Y G D V L Q I R I G S T P V L V L S R L D T I R Q A L V R Q G D - D F K G R P	109

HAU:	121–180	
CYP7A1	H F A T S A K A F - - - G H R S I - - D P M D G N T T E N I N D T F I K T L Q G H A L - - - - - - - - - - - - - - - - N	139
CYP3A4	P F G P V G F M K - - - S - - - - A I S I A E D E E W K R L R S L L S P T F T - - - S G K - - - - - - L K E M V P I I A	150
CYP3A5	S L G P V G F M K - - - S - - - - A I S L A E D E E W K R I R S L L S P T F T - - - S G K - - - - - - L K E M F P I I A	150
CYP2D6	P V P I T Q I L G F G P R S Q G V - F L A R Y G P A W R E Q R R F S V S T L R N L G L G K - - - - - - - K S L E Q W V T	154
CYP2E1	D L P A F - H A H - - - R D R G I - - I F N N G P T W K D I R R F S L T T L R N Y G M G K - - - - - - - Q G N E S R I Q	148
CYP2C8	N S P I S Q R I T - - - K G L G I - - I S S N G K R W K E I R R F S L T T L R N F G M G K - - - - - - - R S I E D R V Q	146
CYP2C18	S F P V A E K V N - - - K G L G I - - L F S N G K R W K E I R R F C L M T L R N F G M G K - - - - - - - R S I E D R V Q	146
CYP2C9	I F P L A E R A N - - - R G F G I - - V F S N G K K W K E I R R F S L M T L R N F G M G K - - - - - - - R S I E D R V Q	146

CYP2C19 H F P L A E R A N - - - R G F G I - - V F S N G K R W K E I R R F S L M T L R N F G M G K - - - - - - - R S I E D R V Q 146
CYP2B6 K I A M V D P F F - - - R G Y G V - - I F A N G N R W K V L R R F S V T T M R D F G M G K - - - - - - - R S V E E R I Q 147
CYP2A6 E Q A T F D W V F - - - K G Y G V - - V F S N G E R A K Q L R R F S I A T L R D F G V G K - - - - - - - R G I E E R I Q 150
CYP2A13 E Q A T F D W L F - - - K G Y G V - - A F S N G E R A K Q L R R F S I A T L R G F G V G K - - - - - - - R G I E E R I Q 150
CYP1B1 A F A S F R V V S - - - G G R S M A F G H Y S - E H W K V Q R R A A H S M M R N F F T R Q - - - P R S R Q V L E G H V L 171
CYP1A1 D L Y T F T L I S - - - N G Q S M S F S P D S G P V W A A R R R L A Q N G L K S F S I A S D P A S S T S C Y L E E H V S 164
CYP1A2 D L Y T S T L I T - - - D G Q S L T F S T D S G P V W A A R R R L A Q N A L N T F S I A S D P A S S S S C Y L E E H V S 166

HAU: 181 182 183 184 185 186 187 188 189 190 191 192 193 194 195 196 197 198 199 200 201 202 203 204 205 206 207 208 209 210 211 212 213 214 215 216 217 218 219 220 221 222 223 224 225 226 227 228 229 230 231 232 233 234 235 236 237 238 239 240
CYP7A1 S L T E S M M E N L Q R I M R - - P P V S S N S K T A A W V T E G M Y S F C Y R V M F E A G Y L T I F G R D L T R R D T 197
CYP3A4 Q Y G D V L V R N L R R E A E T G K P V T L K D V F G A Y S M D V I T S T - - - - - - - - - - - - S F G V N I D S L N N 198
CYP3A5 Q Y G D V L V R N L R R E A E K G K P V T L K D I F G A Y S M D V I T G T - - - - - - - - - - - - S F G V N I D S L N N 198
CYP2D6 E E A A C L C A A F A N H - - S G R P F R P N G L L D K A V S N V I A S L - - - - - - - - - - - - T C G R R F E Y D D P 200
CYP2E1 R E A H F L L E A L R K T - - Q G Q P F D P T F L I G C A P C N V I A D I - - - - - - - - - - - - L F R K H F D Y N D E 194
CYP2C8 E E A H C L V E E L R K T - - K A S P C D P T F I L G C A P C N V I C S V - - - - - - - - - - - - V F Q K R F D Y K D Q 192
CYP2C18 E E A R C L V E E L R K T - - N A S P C D P T F I L G C A P C N V I C S V - - - - - - - - - - - - I F H D R F D Y K D Q 192
CYP2C9 E E A R C L V E E L R K T - - K A S P C D P T F I L G C A P C N V I C S I - - - - - - - - - - - - I F H K R F D Y K D Q 192
CYP2C19 E E A R C L V E E L R K T - - K A S P C D P T F I L G C A P C N V I C S I - - - - - - - - - - - - I F Q K R F D Y K D Q 192
CYP2B6 E E A Q C L I E E L R K S - - K G A L M D P T F L F Q S I T A N I I C S I - - - - - - - - - - - - V F G K R F H Y Q D Q 193
CYP2A6 E E A G F L I D A L R G T - - G G A N I D P T F F L S R T V S N V I S S I - - - - - - - - - - - - V F G D R F D Y K D K 196
CYP2A13 E E A G F L I D A L R G T - - H G A N I D P T F F L S R T V S N V I S S I - - - - - - - - - - - - V F G D R F D Y E D K 196
CYP1B1 S E A R E L V A L L V R G S A D G A F L D P R P L T V V A V A N V M S A V - - - - - - - - - - - - C F G C R Y S H D D P 219
CYP1A1 K E A E V L I S T L Q E L M A G P G H F N P Y R Y V V V S V T N V I C A I - - - - - - - - - - - - C F G R R Y D H N H Q 212
CYP1A2 K E A K A L I S R L Q E L M A G P G H F D P Y N Q V V V S V A N V I G A M - - - - - - - - - - - - C F G Q H F P E S S D 214

HAU: 241 242 243 244 245 246 247 248 249 250 251 252 253 254 255 256 257 258 259 260 261 262 263 264 265 266 267 268 269 270 271 272 273 274 275 276 277 278 279 280 281 282 283 284 285 286 287 288 289 290 291 292 293 294 295 296 297 298 299 300
CYP7A1 Q K A H I L N N L D N F K Q F - - - - - - - - - - D K V F P A L V A G L P - - - I H M F R T A H N A R E K L A E S L R H 244
CYP3A4 P Q D P F V E N T K K L L R F D F L D P - F F L S I T V F P F L I P I L E V L N I - - - - - - C V F P R E V T N F L R K 251
CYP3A5 P Q D P F V E S T K K F L K F G F L D P - L F L S I I L F P F L T P V F E A L N V - - - - - - S L F P K D T I N F L S K 251
CYP2D6 R F L R L L D L A Q E G L K E - - E S G F L R E V L N A V P V L L - H I P A L A G K V L R F Q K - - - - A F L T Q L D E 253
CYP2E1 K F L R L M Y L F N E N F H L - - L S T P W L Q L Y N N F P S F L H Y L P G S H R K V I K N V A - - - - E V K E Y V S E 248
CYP2C8 N F L T L M K R F N E N F R I - - L N S P W I Q V C N N F P L L I D C F P G T H N K V L K N V A - - - - L T R S Y I R E 246
CYP2C18 R F L N L M E K F N E N L R I - - L S S P W I Q V C N N F P A L I D Y L P G S H N K I A E N F A - - - - Y I K S Y V L E 246
CYP2C9 Q F L N L M E K L N E N I K I - - L S S P W I Q I C N N F S P I I D Y F P G T H N K L L K N V A - - - - F M K S Y I L E 246
CYP2C19 Q F L N L M E K L N E N I R I - - V S T P W I Q I C N N F P T I I D Y F P G T H N K L L K N L A - - - - F M E S D I L E 246
CYP2B6 E F L K M L N L F Y Q T F S L - - I S S V F G Q L F E L F S G F L K Y F P G A H R Q V Y K N L Q - - - - E I N A Y I G H 247
CYP2A6 E F L S L L R M M L G I F Q F - - T S T S T G Q L Y E M F S S V M K H L P G P Q Q Q A F Q L L Q - - - - G L E D F I A K 250
CYP2A13 E F L S L L R M M L G S F Q F - - T A T S T G Q L Y E M F S S V M K H L P G P Q Q Q A F K E L Q - - - - G L E D F I A K 250
CYP1B1 E F R E L L S H N E E F G R T - - V G A - - - G S L V D V M P W L Q Y F P N P V R T V F R E F E Q L N R N F S N F I L D 274
CYP1A1 E L L S L V N L N N N F G E V - - V G S - - - G N P A D F I P I L R Y L P N P S L N A F K D L N - - - E K F Y S F M Q K 264
CYP1A2 E M L S L V K N T H E F V E T - - A S S - - - G N P L D F F P I L R Y L P N P A L Q R F K A F N - - - Q R F L W F L Q K 266

HAU: 301 302 303 304 305 306 307 308 309 310 311 312 313 314 315 316 317 318 319 320 321 322 323 324 325 326 327 328 329 330 331 332 333 334 335 336 337 338 339 340 341 342 343 344 345 346 347 348 349 350 351 352 353 354 355 356 357 358 359 360
CYP7A1 E N L Q K R E S I - - - - - - - - S E L I S - - - L R M F L - - - - - - N D T L S T F D D L E K A K T H L V V L W A S Q 287

CYP3A4 S V K R M K E S R L E D T Q K H R V D F L Q L M I D - S Q N S K E - - - T E S H K A L S D L E L V A Q S I I F I F A G Y 307
CYP3A5 S V N R M K K S R L N D K Q K H R L D F L Q L M I D - S Q N S K E - - - T E S H K A L S D L E L A A Q S I I F I F A G Y 307
CYP2D6 L L T E H R M T W - - D P A Q P P R D L T E A F L A E M E K A K G - - - - N P E S S F N D E N L R I V V A D L F S A G M 307
CYP2E1 R V K E H H Q S L - - D P N - C P R D L T D C L L V E M E K E K H - - - - S A E R L Y T M D G I T V T V A D L F F A G T 301
CYP2C8 K V K E H Q A S L - - D V N - N P R D F I D C F L I K M E Q E K D - - - - N Q K S E F N I E N L V G T V A D L F V A G T 299
CYP2C18 R I K E H Q E S L - - D M N - S A R D F I D C F L I K M E Q E K H - - - - N Q Q S E F T V E S L I A T V T D M F G A G T 299
CYP2C9 K V K E H Q E S M - - D M N - N P Q D F I D C F L M K M E K E K H - - - - N Q P S E F T I E S L E N T A V D L F G A G T 299
CYP2C19 K V K E H Q E S M - - D I N - N P R D F I D C F L I K M E K E K Q - - - - N Q Q S E F T I E N L V I T A A D L L G A G T 299
CYP2B6 S V E K H R E T L - - D P S - A P K D L I D T Y L L H M E K E K S - - - - N A H S E F S H Q N L N L N T L S L F F A G T 300
CYP2A6 K V E H N Q R T L - - D P N - S P R D F I D S F L I R M Q E E E K - - - - N P N T E F Y L K N L V M T T L N L F I G G T 303
CYP2A13 K V E H N Q R T L - - D P N - S P R D F I D S F L I R M Q E E E K - - - - N P N T E F Y L K N L V M T T L N L F F A G T 303
CYP1B1 K F L R H C E S L - - R P G A A P R D M M D A F I L S A E K K A A G D S H G G G A R L D L E N V P A T I T D I F G A S Q 332
CYP1A1 M V K E H Y K T F - - E K G - H I R D I T D S L I E H C Q E K Q L - - D E N A N V Q L S D E K I I N I V L D L F G A G F 319
CYP1A2 T V Q E H Y Q D F - - D K N - S V R D I T G A L F K H S K K G P R - - - - A S G N L I P Q E K I V N L V N D I F G A G F 319

HAU: 361 362 363 364 365 366 367 368 369 370 371 372 373 374 375 376 377 378 379 380 381 382 383 384 385 386 387 388 389 390 391 392 393 394 395 396 397 398 399 400 401 402 403 404 405 406 407 408 409 410 411 412 413 414 415 416 417 418 419 420
CYP7A1 A N T I P A T F W S L F Q M I R N P E A M K A A T E E V K R T L E N A G Q K V S L E G N P I C L S Q A E L N D L P V L D 347
CYP3A4 E T T S S V L S F I M Y E L A T H P D V Q Q K L Q E E I D A V L P N K A P - - - - - - - - - P T - - Y D T V L Q M E Y L D 357
CYP3A5 E T T S S V L S F T L Y E L A T H P D V Q Q K L Q K E I D A V L P N K A P - - - - - - - - - P T - - Y D A V V Q M E Y L D 357
CYP2D6 V T T S T T L A W G L L L M I L H P D V Q R R V Q Q E I D D V I G Q V R R - - - - - - - - - P E - - M G D Q A H M P Y T T 357
CYP2E1 E T T S T T L R Y G L L I L M K Y P E I E E K L H E E I D R V I G P S R I - - - - - - - - - P A - - I K D R Q E M P Y M D 351
CYP2C8 E T T S T T L R Y G L L L L L K H P E V T A K V Q E E I D H V I G R H R S - - - - - - - - - P C - - M Q D R S H M P Y T D 349
CYP2C18 E T T S T T L R Y G L L L L L K Y P E V T A K V Q E E I E C V V G R N R S - - - - - - - - - P C - - M Q D R S H M P Y T D 349
CYP2C9 E T T S T T L R Y A L L L L L K H P E V T A K V Q E E I E R V I G R N R S - - - - - - - - - P C - - M Q D R S H M P Y T D 349
CYP2C19 E T T S T T L R Y A L L L L L K H P E V T A K V Q E E I E R V I G R N R S - - - - - - - - - P C - - M Q D R G H M P Y T D 349
CYP2B6 E T T S T T L R Y G F L L M L K Y P H V A E R V Y R E I E Q V I G P H R P - - - - - - - - - P E - - L H D R A K M P Y T E 350
CYP2A6 E T V S T T L R Y G F L L L M K H P E V E A K V H E E I D R V I G K N R Q - - - - - - - - - P K - - F E D R A K M P Y M E 353
CYP2A13 E T V S T T L R Y G F L L L M K H P E V E A K V H E E I D R V I G K N R Q - - - - - - - - - P K - - F E D R A K M P Y T E 353
CYP1B1 D T L S T A L Q W L L L L F T R Y P D V Q T R V Q A E L D Q V V G R D R L - - - - - - - - - P C - - M G D Q P N L P Y V L 382
CYP1A1 D T V T T A I S W S L M Y L V M N P R V Q R K I Q E E L D T V I G R S R R - - - - - - - - - P R - - L S D R S H L P Y M E 369
CYP1A2 D T V T T A I S W S L M Y L V T K P E I Q R K I Q K E L D T V I G R E R R - - - - - - - - - P R - - L S D R P Q L P Y L E 369

HAU: 421 422 423 424 425 426 427 428 429 430 431 432 433 434 435 436 437 438 439 440 441 442 443 444 445 446 447 448 449 450 451 452 453 454 455 456 457 458 459 460 461 462 463 464 465 466 467 468 469 470 471 472 473 474 475 476 477 478 479 480
CYP7A1 S I I K E S L R L S S A - S L N I R T A K E D F T L H L E D G S Y N I R K D D I I A L Y P Q L M H L D P E I Y P D P L T 406
CYP3A4 M V V N E T L R L F P - I A M R L E - - - - R V C K K D V E I N G M F I P K G V V V M I P S Y A L H R D P K Y W T E P E K 413
CYP3A5 M V V N E T L R L F P - V A I R L E - - - - R T C K K D V E I N G V F I P K G S M V V I P T Y A L H H D P K Y W T E P E E 413
CYP2D6 A V I H E V Q R F G D I V P L G V T - - - - H M T S R D I E V Q G F R I P K G T T L I T N L S S V L K D E A V W E K P F R 414
CYP2E1 A V V H E I Q R F I T L V P S N L P - - - - H E A T R D T I F R G Y L I P K G T V V V P T L D S V L Y D N Q E F P D P E K 408
CYP2C8 A V V H E I Q R Y S D L V P T G V P - - - - H A V T T D T K F R N Y L I P K G T T I M A L L T S V L H D D K E F P N P N I 406
CYP2C18 A V V H E I Q R Y I D L L P T N L P - - - - H A V T C D V K F K N Y L I P K G T T I I T S L T S V L H N D K E F P N P E M 406
CYP2C9 A V V H E V Q R Y I D L L P T S L P - - - - H A V T C D I K F R N Y L I P K G T T I L I S L T S V L H D N K E F P N P E M 406
CYP2C19 A V V H E V Q R Y I D L I P T S L P - - - - H A V T C D V K F R N Y L I P K G T T I L T S L T S V L H D N K E F P N P E M 406
CYP2B6 A V I Y E I Q R F S D L L P M G V P - - - - H I V T Q H T S F R G Y I I P K D T E V F L I L S T A L H D P H Y F E K P D A 407
CYP2A6 A V I H E I Q R F G D V I P M S L A - - - - R R V K K D T K F R D F F L P K G T E V Y P M L G S V L R D P S F F S N P Q D 410
CYP2A13 A V I H E I Q R F G D M L P M G L A - - - - H R V N K D T K F R D F F L P K G T E V F P M L G S V L R D P R F F S N P R D 410

CYP1B1 A F L Y E A M R F S S F V P V T I P - - - H A T T A N T S V L G Y H I P K D T V V F V N Q W S V N H D P L K W P N P E N 439
CYP1A1 A F I L E T F R H S S F V P F T I P - - - H S T T R D T S L K G F Y I P K G R C V F V N Q W Q I N H D Q K L W V N P S E 426
CYP1A2 A F I L E T F R H S S F L P F T I P - - - H S T T R D T T L N G F Y I P K K C C V F V N Q W Q V N H D P E L W E D P S E 426

HAU: 481 482 483 484 485 486 487 488 489 490 491 492 493 494 495 496 497 498 499 500 501 502 503 504 505 506 507 508 509 510 511 512 513 514 515 516 517 518 519 520 521 522 523 524 525 526 527 528 529 530 531 532 533 534 535 536 537 538 539 540
CYP7A1 F K Y D R Y L D E N G K T K T T F Y C N G L K L K Y Y Y M P F G S G A T I C P G R L F A I H E I K Q F L I L M L S Y F E 466
CYP3A4 F L P E R F S K K N K D - - - - - - - - - N I D P Y I Y T P F G S G P R N C I G M R F A L M N M K L A L I R V L Q N F S 464
CYP3A5 F R P E R F S K K - K D - - - - - - - - - S I D P Y I Y T P F G T G P R N C I G M R F A L M N M K L A L I R V L Q N F S 463
CYP2D6 F H P E H F L D A Q G H - - - - - - - - - F V K P E A F L P F S A G R R A C L G E P L A R M E L F L F F T S L L Q H F S 465
CYP2E1 F K P E H F L N E N G K - - - - - - - - - F K Y S D Y F K P F S T G K R V C A G E G L A R M E L F L L L C A I L Q H F N 459
CYP2C8 F D P G H F L D K N G N - - - - - - - - - F K K S D Y F M P F S A G K R I C A G E G L A R M E L F L F L T T I L Q N F N 457
CYP2C18 F D P G H F L D K S G N - - - - - - - - - F K K S D Y F M P F S A G K R M C M G E G L A R M E L F L F L T T I L Q N F N 457
CYP2C9 F D P H H F L D E G G N - - - - - - - - - F K K S K Y F M P F S A G K R I C V G E A L A G M E L F L F L T S I L Q N F N 457
CYP2C19 F D P R H F L D E G G N - - - - - - - - - F K K S N Y F M P F S A G K R I C V G E G L A R M E L F L F L T F I L Q N F N 457
CYP2B6 F N P D H F L D A N G A - - - - - - - - - L K K T E A F I P F S L G K R I C L G E G I A R A E L F L F F T T I L Q N F S 458
CYP2A6 F N P Q H F L N E K G Q - - - - - - - - - F K K S D A F V P F S I G K R N C F G E G L A R M E L F L F F T T V M Q N F R 461
CYP2A13 F N P Q H F L D K K G Q - - - - - - - - - F K K S D A F V P F S I G K R Y C F G E G L A R M E L F L F F T T I M Q N F R 461
CYP1B1 F D P A R F L D K D G - L I - - - - - - N K D L T S R V M I F S V G K R R C I G E E L S K M Q L F L F I S I L A H Q C D 492
CYP1A1 F L P E R F L T P D G - A I - - - - - - D K V L S E K V I I F G M G K R K C I G E T I A R W E V F L F L A I L L Q R V E 479
CYP1A2 F R P E R F L T A D G T A I - - - - - - N K P L S E K M M L F G M G K R R C I G E V L A K W E I F L F L A I L L Q Q L E 480

HAU: 541 542 543 544 545 546 547 548 549 550 551 552 553 554 555 556 557 558 559 560 561 562 563 564 565 566 567 568 569 570 571 572 573 574 575 576 577 578 579 580 581 582 583 584 585 586 587 588 589 590 591 592 593 594 595 596 597 598 599 600
CYP7A1 L E L I E G Q A K C P P L D Q S R A G L G I L P P L N D I E F K Y K F K H - L - 504
CYP3A4 F K P C K E T - Q I P - L K L S L G G L - - L Q P E K P V V L K V E S R D - - - - - - - - - - G T V S G A - - - - - - - 503
CYP3A5 F K P C K E T - Q I P - L K L D T Q G L - - L Q P E K P I V L K V D S R D - - - - - - - - - - G T L S G E - - - - - - - 502
CYP2D6 F S V P T G Q - P R P S - H H G V F A F L V S P S P Y E L C A V P R - 497
CYP2E1 L K P L V D P - K D I D L S P I H I G F G C I P P R Y K L C V I P R S - 493
CYP2C8 L K S V D D L - K N L N T T A V T K G I V S L P P S Y Q I C F I P V - 490
CYP2C18 L K S Q V D P - K D I D I T P I A N A F G R V P P L Y Q L C F I P V - 490
CYP2C9 L K S L V D P - K N L D T T P V V N G F A S V P P F Y Q L C F I P V - 490
CYP2C19 L K S L I D P - K D L D T T P V V N G F A S V P P F Y Q L C F I P V - 490
CYP2B6 M A S P V A P - E D I D L T P Q E C G V G K I P P T Y Q I R F L P R - 491
CYP2A6 L K S S Q S P - K D I D V S P K H V G F A T I P R N Y T M S F L P R - 494
CYP2A13 F K S P Q S P - K D I D V S P K H V G F A T I P R N Y T M S F L P R - 494
CYP1B1 F R A N P N E - P A - - - - - - - - - - - K M N F S Y G L T I K P K S F K - V N V T L R E S M E L L D S A V Q N L Q A K 539
CYP1A1 F S V P L G V - K V - - - - - - - - - - - D M T P I Y G L T M K H A C C E H F Q M Q L R S - - - - - - - - - - - - - - - 512
CYP1A2 F S V P P G V - K V - - - - - - - - - - - D L T P I Y G L T M K H A R C E H V Q A R L R F S I N - - - - - - - - - - - - 516

HAU: 601 602 603 604
CYP7A1 - - - - 504
CYP3A4 - - - - 503
CYP3A5 - - - - 502
CYP2D6 - - - - 497
CYP2E1 - - - - 493

CYP2C8	-	-	-	-	490
CYP2C18	-	-	-	-	490
CYP2C9	-	-	-	-	490
CYP2C19	-	-	-	-	490
CYP2B6	-	-	-	-	491
CYP2A6	-	-	-	-	494
CYP2A13	-	-	-	-	494
CYP1B1	E	T	C	Q	543
CYP1A1	-	-	-	-	512
CYP1A2	-	-	-	-	

Fig. S1. The comparison of the amino-acid sequences of 15 CYP450 enzymes considered in the current study. The variations collected in Table xxx (main manuscript) and discussed are highlighted in yellow.

	CP7A1	CP3A4	CP3A5	CP2D6	CP2E1	CP2C8	CP2C18	CP2C9	CP2C19	CP2B6	CP2A6	CP2A13	CP1B1	CP1A1	CP1A2
CP7A1	100.00	20.85	19.10	17.62	18.46	18.81	19.69	19.91	20.35	20.31	16.89	17.32	17.25	18.86	19.08
CP3A4	20.85	100.00	84.26	21.06	24.09	25.11	24.89	24.46	24.89	23.50	24.26	23.83	18.56	20.89	21.59
CP3A5	19.10	84.26	100.00	21.54	23.50	25.16	23.87	24.30	24.09	23.55	21.96	21.32	19.21	21.78	22.90
CP2D6	17.62	21.06	21.54	100.00	38.78	40.16	38.93	39.14	39.55	39.88	35.16	36.38	34.10	30.33	30.06
CP2E1	18.46	24.09	23.50	38.78	100.00	56.65	56.85	57.06	57.06	46.01	47.15	47.15	28.57	29.98	29.08
CP2C8	18.81	25.11	25.16	40.16	56.65	100.00	77.14	77.96	78.16	49.69	48.98	50.82	31.71	30.38	28.42
CP2C18	19.69	24.89	23.87	38.93	56.85	77.14	100.00	81.84	80.82	47.43	48.98	50.00	31.71	29.32	27.37
CP2C9	19.91	24.46	24.30	39.14	57.06	77.96	81.84	100.00	91.43	48.05	49.18	50.00	31.71	30.59	28.63
CP2C19	20.35	24.89	24.09	39.55	57.06	78.16	80.82	91.43	100.00	47.84	51.02	51.43	30.87	29.75	27.16
CP2B6	20.31	23.50	23.55	39.88	46.01	49.69	47.43	48.05	47.84	100.00	51.53	53.77	31.65	29.26	27.31
CP2A6	16.89	24.26	21.96	35.16	47.15	48.98	48.98	49.18	51.02	51.53	100.00	93.52	28.30	31.38	28.60
CP2A13	17.32	23.83	21.32	36.38	47.15	50.82	50.00	50.00	51.43	53.77	93.52	100.00	29.56	32.01	29.65
CP1B1	17.25	18.56	19.21	34.10	28.57	31.71	31.71	31.71	30.87	31.65	28.30	29.56	100.00	39.45	38.43
CP1A1	18.86	20.89	21.78	30.33	29.98	30.38	29.32	30.59	29.75	29.26	31.38	32.01	39.45	100.00	72.55
CP1A2	19.08	21.59	22.90	30.06	29.08	28.42	27.37	28.63	27.16	27.31	28.60	29.65	38.43	72.55	100.00

Fig. S2. The percent identity matrix created for amino-acid sequences of 15 CYP450 enzymes considered in the current study. The www.ebi.ac.uk/jdispatcher/msa/muscle online server and the Clustal 2.1 algorith were used.
